# Prediction of hERG inhibition of drug discovery compounds using biomimetic HPLC measurements

**DOI:** 10.5599/admet.995

**Published:** 2021-06-06

**Authors:** Chrysanthos Stergiopoulos, Fotios Tsopelas, Klara Valko

**Affiliations:** 1 Laboratory of Inorganic and Analytical Chemistry, School of Chemical Engineering, National Technical University of Athens; 2 Bio-Mimetic Chromatography Ltd. Stevenage, Herts, United Kingdom

**Keywords:** Cardiotoxicity, Proarrhythmia, QT Prolongation, Torsades de Pointes, hERG inhibition, IAM binding, AGP binding

## Abstract

The major causes of failure of drug discovery compounds in clinics are the lack of efficacy and toxicity. To reduce late-stage failures in the drug discovery process, it is essential to estimate early the probability of adverse effects and potential toxicity. Cardiotoxicity is one of the most often observed problems related to a compound’s inhibition of the hERG channel responsible for the potassium cation flux. Biomimetic HPLC methods can be used for the early screening of a compound’s lipophilicity, protein binding and phospholipid partition. Based on the published hERG pIC50 data of 90 marketed drugs and their measured biomimetic properties, a model has been developed to predict the hERG inhibition using the measured binding of compounds to alpha-1-acid-glycoprotein (AGP) and immobilised artificial membrane (IAM). A representative test set of 16 compounds was carefully selected. The training set, involving the remaining compounds, served to establish the linear model. The mechanistic model supports the hypothesis that compounds have to traverse the cell membrane and bind to the hERG ion channel to cause the inhibition. The AGP and the hERG ion channel show structural similarity, as both bind positively charged compounds with strong shape selectivity. In contrast, a good IAM partition is a prerequisite for cell membrane traversal. For reasons of comparison, a corresponding model was derived by replacing the measured biomimetic properties with calculated physicochemical properties. The model established with the measured biomimetic binding properties proved to be superior and can explain over 70% of the variance of the hERG pIC50 values.

## Introduction

It has been recognised that the physicochemical properties of drug candidates can be related to the late-stage attrition of compounds in the drug development process. The early problems with bioavailability and absorption have been successfully improved by optimising solubility and permeability [1]. Recently, toxicity and the lack of efficacy have been identified as the major cause of compound attrition in clinics. Together, preclinical toxicity and adverse events account for approximately one-third of all attrition cases [2].

Cardiotoxicity is one of the major causes of concern during clinical trials together with liver and central nervous system (CNS) toxicity [3]. It accounts for approximately 27 % of drug development failures, and it does not seem to be restricted to specific high-risk therapeutic areas [4]. One particular focus of cardiovascular adverse effects has been drug-induced arrhythmia or “proarrhythmia” as a consequence of an increased recognition of a relationship between drug-induced QT interval prolongation and Torsades de Pointes (TdP) [5]. TdP is a dangerous type of proarrhythmia, described as a rare ventricular tachycardia with potential sudden cardiac death, which has led to approximately one-third of all drug withdrawals between 1990 and 2006 [4]. Furthermore, 15 % of drugs still on the market can cause QT prolongation, and 4 % are associated with TdP arrhythmia risk. Therefore, it is important to recognize a compound’s cardiotoxicity potential early in the drug discovery process, not only because of the associated loss of human life or health, but also because of the enormous financial loss in investment and future revenue potential [6].

The cardiac action potential is regulated by the electrical current flows of ions across cardiomyocyte membranes. Many drugs can bind to ion channels, block ionic flow and disrupt the regulation of the action potential [7]. Upon blockade, the action potential will rest longer, which results in an increased duration of the relative QT interval that can be observed in electrocardiograph (ECG) traces. Disturbing the QT interval may lead to instability in the heart rhythm [8]. Patients with long QT syndrome (LQTS) exhibit a significant predisposition for the TdP type’s cardiac arrhythmia [9]. A prolongation of the cardiac action potential and the QT interval has been associated with loss of function or drug-trapping inside the central cavity of the Kv11.1 [10] potassium channel, which is encoded by hERG (human Ether-a-go-go Related Gene) and carries the rapid delayed rectifier potassium current (*I*_Kr_) [7,11]. This channel has a tetrameric structure formed by co-assembly of four identical subunits, each composed of six helical transmembrane domains (denoted S1–S6). The S4 domain contains six positive charges, typical for voltage-gated K^+^ channels [12]. The channel pore is asymmetrical, and its dimensions change depending on its state (open-closed-inactivated). The hERG channel has been shown to interact with a wide range of drugs owing to the unique shape of the ligand-binding site, its hydrophobic character and the large vestibule of the channel [13,14]. The risk tolerance for QT prolongation may vary significantly depending on the dose and indication of the drug. Documented hERG-blocking activity reduces the value of a molecule, as it increases the risk of clinical failure. It has also been estimated that about 60 % of drugs in development exhibit hERG block [11].

Various attempts have been made to predict the hERG inhibition potential of drugs *in silico* to avoid the synthesis of risky molecules [15]. When studying therapeutic areas and the safety margins regarding the free therapeutic plasma concentration of drugs [16], it was found that a wide variety of drugs, including antiarrhythmic, antibacterial, antipsychotic and pain-killer drugs showed potential risk. As toxicity, just like potency, is dose-dependent, it is essential to relate the hERG inhibitory concentration to the drugs’ free therapeutic plasma concentration. It was found that a less than 30-fold difference between the therapeutic and inhibitory concentration indicates a high risk. Redfern et al. [16] also investigated the relative value of preclinical cardiac electrophysiology data (*in vitro* and *in vivo*) for predicting the risk of TdP in drug clinical use. *In vivo*, telemetry experiments in non-rodents (typically dogs) are the ultimate preclinical test for cardiotoxicity. However, its high cost severely limits its use at the earlier discovery stage [17]. *In vitro* voltage-clamp techniques are widely used to provide real-time mechanistic information on ion channels [18]. The experiments are performed in mammalian cells transfected with the gene for hERG. The overwhelming majority of predictive hERG models have been built using mammalian patch clamp data. Techniques such as fluorescence-based assays with cells transfected with hERG and radioligand (typically dofetilide or MK-499) displacement assays [17] have also been successfully used. Since the success of any model building depends on the quality of the biological data, it was important to carefully select reliable and informative cardiotoxicity data for a wide variety of drugs in order to develop a continuous model. As the determination of the half-maximal inhibitory concentration (IC_50_) value requires measurements of inhibitory activities at multiple concentrations, the IC_50_ information was considered more reliable, and was selected over the inhibition type entries for positive/negative classification. Therefore, IC_50_ of drugs and their log unit values (pIC_50_) in response to hERG were collected from the literature.

Certain physicochemical properties of molecules have been recognized as early indicators of potential problems with early drug discovery compounds [19]. Besides lipophilicity [20], solubility [21] and permeability, biomimetic properties such as protein [22] and phospholipid binding [23] can be measured at the early stages of the drug discovery process [24]. The chromatographic technique provides an automated, high throughput and reliable measurement of important properties of the drug discovery compounds [25] that can be used to estimate later stage *in vivo* properties of compounds such as the volume of distribution, the unbound volume of distribution [26] and the drug efficiency [27]. Measurements can also estimate cell membrane partition and skin penetration of compounds based on chromatographic principles [28,29]. Various toxicity indicators have already been related to a compound’s physicochemical properties, including hERG inhibition and hepatotoxicity [30]. The toxicity potential of compounds has been studied using the immobilised artificial membrane (IAM) chromatography [31]. In this work, several chromatography-based techniques were investigated to search for the properties of the compounds that could be used to predict their toxicity, with special emphasis on cardiotoxicity.

In this study, hERG pIC_50_ data from a set of 90 diverse marketed drugs from a wide range of therapeutic areas and with different physicochemical properties were correlated with their measured biomimetic properties. The measurement of the biomimetic properties of the available drugs was conducted in our laboratories. Generic gradient HPLC methods were used to determine the chromatographic hydrophobicity Index (CHI) [32,33] using mobile phases at three different pH values. The protein binding of the compounds was measured using immobilised human serum albumin (HSA) [22], and alpha-1-acid-glycoprotein (AGP) stationary phases [34]. The phospholipid-binding was measured using the immobilised artificial membrane (IAM) stationary phase [23]. The aim was to establish relationships between the cardiotoxicity potential and the biomimetic binding properties of the drugs and to evaluate their predictive performance.

## Experimental

The drugs were obtained from Sigma-Aldrich (Merck) and dissolved in dimethylsulfoxide (DMSO) at 10 mM concentration. The 10 μL stock solutions were diluted down to 100 μL before injecting them onto an Agilent 1100 HPLC system.

### CHI lipophilicity measurements

The Chromatographic Hydrophobicity Index (CHI) was measured using the compounds’ calibrated gradient retention times obtained from an Agilent 1100 HPLC fitted with a Gemini NX-C-18 column (Phenomenex Ltd Macclesfield, UK) with dimensions of 50 x 3 mm and 5 μm particle size. The mobile phase A was either 0.01 M formic acid (pH 2.6), a 50 mM ammonium acetate buffer with an adjusted pH of 7.4 or a 50 mM ammonium acetate buffer with an adjusted pH of 10.5. The mobile phase B was 100 % acetonitrile. The flow rate was 1.0 mL/min, with starting mobile phases of 0.01M formic acid (pH 2.6), 50 mM ammonium acetate adjusted to pH 7.4, and 50 mM ammonium acetate adjusted to pH 10.5 to determine the lipophilicity of the compounds at acidic, neutral and alkaline pHs, respectively. An acetonitrile linear gradient was used from 0 to 100 %. The acetonitrile concentration reached 100 % in 3.5 min. The 100 % acetonitrile mobile phase was maintained for an additional 1 min before it was returned to 0 % at 4.7 min. The gradient run cycle time was 6 min, with an additional equilibration time of 1 min before the next injection. The standard deviation in the retention time measurements is ±0.005 min from repeated injections. The retention time values for a standard set of compounds listed in Table 1 were used to convert the drug retention times to CHI values.

### Measurements of plasma protein binding using Chiralpak HSA and AGP columns

The protein binding measurements were carried out on Chiralpak HSA and Chiralpak AGP columns with dimensions of 3 x 50 mm and 5 μm particle size (Chiral Technologies Europe, France). The mobile phase was 50 mM ammonium acetate adjusted to pH 7.4, with a 1.2 mL/min flow rate. The standard isopropanol (IPA) gradient reached 35 % in 3.5 min, which was maintained for 1 min, before returning to 0 % at 4.7 min. The cycle time was 6 min with an additional 1 min re-equilibration time. The racemic warfarin showed separation of its enantiomers at retention times of 3.58 and 3.77 min. The precision of the retention time measurements was within ±0.01 min. The calibration set of compounds and their literature % binding data which were also converted to log *k* data are shown in Table 2.

### Measurements of phospholipid-binding at pH 7.4 using an IAM column

The phospholipid-binding was measured using an IAM PC.DD2 column with dimensions of 100 x 4.6 mm (Regis Technologies Inc., Morton Grove, IL, USA). The gradient retention times were measured using a 50 mM ammonium acetate mobile phase with the pH adjusted to 7.4. The mobile phase flow rate was 1.5 mL/min. The acetonitrile gradient was applied to reach 90 % in 4.75 min. The 90 % acetonitrile concentration was maintained for an additional 0.5 min (to 5.25 min) and returned to 0 % by 5.5 min. The cycle time was 6 min, plus an additional 1 min equilibration time was applied while the injector prepared for the next injection. The gradient retention times were calibrated with the acetophenone homologues for which the CHI IAM values have been established using isocratic measurements [34]. Table 3 shows the calibration set of compounds and their predetermined CHI IAM values. The CHI Index on the IAM column (CHI IAM) approximates the acetonitrile concentration in the mobile phase when the compound elutes. CHI IAM values above 45 indicate strong phospholipid binding. The CHI IAM values have been converted to log *k* IAM values derived from the CHI IAM values using equation 1. It represents the equivalent value derived from several isocratic measurements with extrapolated log retention factors to 100 % aqueous mobile phase [23]. The log *k* IAM values can be converted to log *K* (IAM) values and show linear relationships with the octanol/water partition coefficients [26]. Equation 2 shows the conversion:


(1)

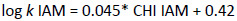




(2)

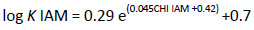



Repeating the retention time measurements provided a standard deviation of ±0.005 min.

### Database search for pIC_50_ values

Assessing the risk of a blockade of the human ether à-go-go related gene potassium channels could greatly facilitate the development of therapeutic compounds and the withdrawal of hazardous marketed drugs. The development of high-throughput automated patch clamp assays has increased the amount of hERG-associated data available in public databases [17]. Integrated databases are now available using the ChEMBL and PubChem public databases. A large integrated database created by Sato et al. [36] has been used in this study. This database curates hERG-related data from *in vitro* assays, such as binding assays (radioligand replacement assay) and electrostatic assays (automated patch-clamp assays), in ChEMBL, PubChem, GOSTAR, NIH Chemical Genomics Center (NCGC) and hERGCentral and integrates them into the largest database about hERG inhibition. IC_50_ values of the compounds and their pIC_50_ values expressed in molar concentrations were carefully searched and collected from this database, which is freely available at https://drugdesign.riken.jp/hERGdb/. Data entries using inequality signs, NULL values and value ranges were excluded. In cases of differences in the reported data for the same compound, mean values were calculated and considered for the model building while outlier values were omitted when the deviation in the results was significant (data points not falling within three standard deviations of the mean).

### Calculated physicochemical properties

ADME Boxes v.3.0 software (Pharma Algorithm) was used to calculate various physicochemical parameters of the investigated compounds such as octanol-water partition (log *P*) and distribution (log *D*) coefficients at the pH values of 7.4, hydrogen bond donor (HBD) and acceptor (HBA) groups, Abraham’s hydrogen bond acidity (A) and basicity (B), total polar surface area (TPSA), molecular weight (MW), as well as the molecular fractions of positively charged (F+), negatively charged (F-) and zwitterionic species (Fz) at pH =7.4.

### Statistical and visualisation software

JMP v13.0 (SAS Institute Inc) and SPSS 23.0 (IBM SPSS Statistics) were used for the statistical calculations and the stepwise regression analysis. For visualisation, Stardrop (Optibrium Ltd) chemically aware visualisation tools were used to create the plots.

## Results and Discussion

Table 4 contains the collected and quality checked pIC_50_ data of the investigated 90 drug molecules with their generic names and the measured biomimetic HPLC data. The drugs used in the training set and test set are listed separately in alphabetical order.

Table 5 contains the calculated physicochemical properties of the investigated compounds. The test set listed separately in alphabetical order in the last part of the table.

### Selection of the test set of compounds

**Figure 1:**
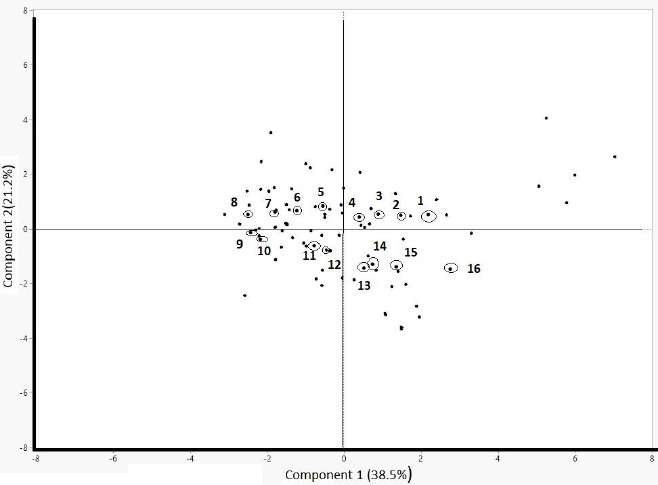
The score plot from the principal component analysis on the calculated properties. Compounds served as the test set are marked in red, as follows: (1) dofetilide, (2) sildenafil, (3) irbesartan, (4) flecainide, (5) ziprasidone, (6) trazodone, (7) fluoxetine, (8) protryptiline, (9) diphenhydramine, (10) desloratanide, (11) spironolactone, (12) metoprolol, (13) lamotrigine, (14) indomethacin, (15) trimethoprim, (16) alfuzosin.

Constructed toxicity models require external validation to prove their predictive ability. Hence, a test set, usually consisting of about 20 % of the entire set, is necessary to evaluate the established models in terms of their predictive performance [37]. For that reason, a principal component analysis using the calculated physicochemical properties of the compounds was performed. By plotting the first two principal components (Figure 1), four compounds from each quadrant were selected by taking into account the compounds’ therapeutic areas to ensure the test set’s diversity. The remaining compounds were used for modelling as the training set. Table 6 shows the therapeutic areas of the compounds selected as the test set.

Using stepwise regression analysis on the training set, a model was built using only the measured properties listed in Table 4. The best model can be described by equation 3:


(3)

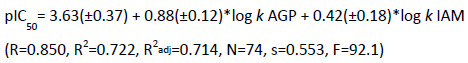



R is the correlation coefficient, N is the number of compounds, s is the standard error of the estimate, F is the Fisher test value. The IAM binding and AGP binding variables are highly significant and showed a week intercorrelation (R^2^=0.42). Therefore, the equation was recalculated using Partial Least Squares regression (PLS) and the same coefficients and intercept were obtained.

The best model using only in silico calculated properties, the molecular weight (MW), the number of H-bond donors (HBD) and acceptor groups (HBA), the polar surface (TPSA), the logarithm of the calculated octanol/water partition coefficient of the neutral form and the combined ionised form of the molecules at pH 7.4 (log *P* and log *D*), calculated fractions of the positive, negative and zwitterionic charges at physiological pH (F+, F-, Fz), and the Abraham H-bond acidity and B-bond basicity parameters (A, B) can be described by equation 4.


(4)

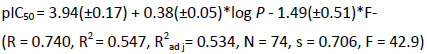



It was found that log *P* is correlated better than log *D* with pIC_50_ and only negatively charged molecular fraction F- stands as statistically significant additional physicochemical parameter next to log *P*. The statistical insignificance of F+ may be attributed to the fact that its positive sign due to ionization counterbalanced with its positive influence to pIC_50_. The models’ statistical parameters are much worse when compared to the model using measured AGP binding (log *k* AGP) data and membrane partition (log *k* IAM) data.

**Figure 2:**
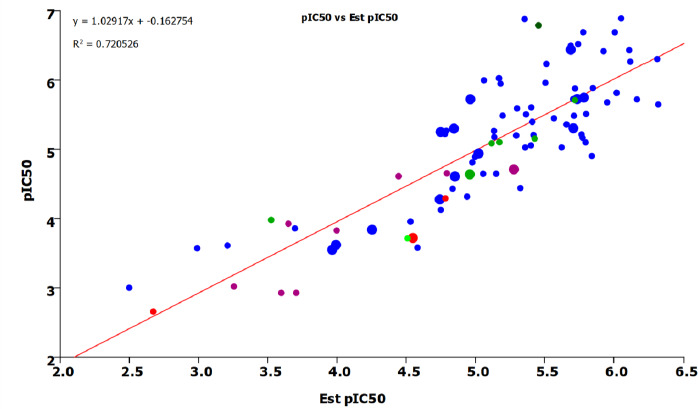
shows the literature hERG pIC_50_ data and the back-calculated pIC_50_ data using **equation 3.** Blue circles mark positively charged compounds at pH 7.4; red circles mark negatively charged compounds at pH7.4. The green circles mark neutral compounds at pH 7.4; green circle’s shade reflects the presence of weak acidic (lighter green) or weak basic (darker green) groups in the molecules; purple circles indicate compounds with zwitterionic character at pH 7.4

The estimated pIC_50_ values of the test set have been calculated using equation 3 and plotted in Figure 2. The test set compounds are marked with larger circles.

**Figure 3.**
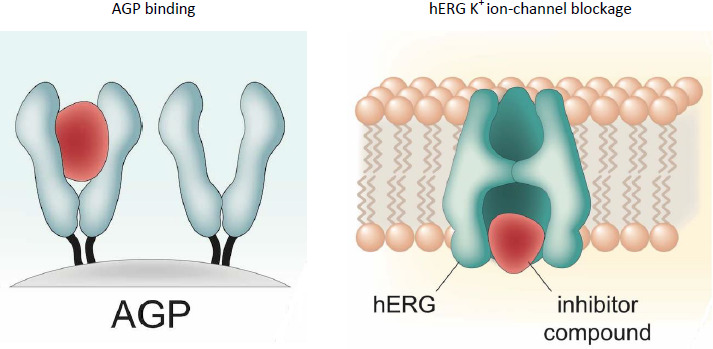
The similarity of the binding region of AGP and hERG potassium ion channels.

It can be seen in Figure 2 that the majority of the positively charged compounds show a pIC_50_ value greater than 5 log units in the *in vitro* hERG experiments. Acidic and zwitterionic compounds show only weak hERG inhibition. It is also interesting to note that the AGP binding data showed a strong correlation with the compounds binding to hERG channel receptors. The explanation for this may lie in the similarity of the two proteins. It was found [38] that the AGP binding site can be featured as a funnel-like structure. The side of the funnel represents a lipophilic region. The funnel’s top width provides a steric hindrance for molecules wider than the funnel, while at the narrow end of the funnel are the negatively charged sialic acid residues that bind the positive charges if they fit into the deep end of the funnel. The structure is illustrated in Figure 3. The IAM binding, which shows the compound’s membrane partition, was also significant in the model, which is not surprising as the ion channel receptor is in the membrane. The compound needs to have high membrane affinity to be able to approach the channel. The positive charge also promotes the binding to the negatively charged surface. Both the AGP and IAM stationary phases show strong shape selectivity, which is also essential to hERG inhibition. Although a wide variety of molecules show high pIC_50_ values the shape of the molecule is very important because of the channel opening’s well-defined size. This fact reduces the power of the *in silico* models if only 2D descriptors are used in the model building. As a result, the 3D description of the molecules would probably enhance the success of *in silico* models.

**Figure 4.**
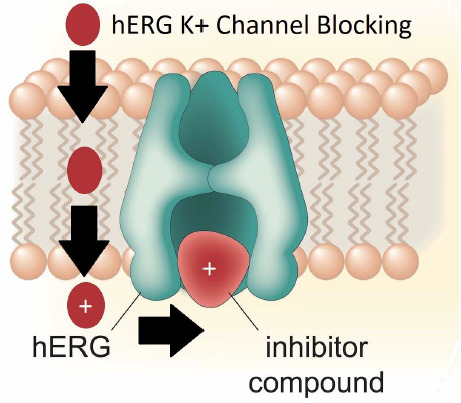
Drug trapping within the K1 channel vestibule.

The steric structure and the negatively charged surface of AGP and the hERG ion channel suggest strong similarities. Compounds that block the channel have to penetrate the cell and have a relatively high concentration in the cell membrane where the potassium ion channel can be found [39]. This explains the importance of the membrane-binding properties in the model, as shown in Figure 4.

Validation of both models was performed by predictions of the 16 compounds included in the test set. The results are presented in Table 7. It can be seen that the prediction of the test set was superior in the case of the model derived with the measured properties, and the residuals did not exceed double the model error (0.693). On the other hand, predictions from the model derived with the calculated properties exhibited much worse residuals in most cases, with the pIC_50_ predictions of irbesartan and lamotrigine exceeding 1 log unit.

## Conclusions

The hERG channel inhibition properties of drugs and drug discovery compounds are an important attribute as compounds with strong binding can cause cardiotoxic side effects during clinical trials. Early recognition of a compound’s hERG inhibition potential is important to avoid the progression of compounds that fail later because of cardiotoxicity.

It has been demonstrated that two biomimetic HPLC property measurements can be used for screening molecules for hERG inhibition potential at an early stage of the drug discovery process. The model is based on the strong similarity between the AGP and hERG channel structures. Both attract positively charged compounds with a significant degree of lipophilicity. Both exhibit steric hindrance depending on the size and shape of the molecule being investigated. The membrane partition is also an important parameter as it reveals the membrane affinity of the compounds where the ion channel receptor is located. It has also been shown that two-dimensional physicochemical descriptors cannot provide an acceptable model for estimating the hERG pIC_50_ of molecules.

## Figures and Tables

**Table 1.** The CHI values of the calibration set of compounds at three pHs [33]. These values were obtained by fitting the isocratically determined CHI values and the gradient retention time values. The standard error ranged from 0.1 to 0.8 CHI values. CHI approximates to the acetonitrile concentration when the compound elutes and can be converted to the octanol/water log *D* scale using CHI log *D* = 0.0525*CHI -1.467 [35].

Compound	CHI at pH 2.6	CHI at pH 7.4	CHI at pH 10.5
Theophylline	17.9	18.4	5.0
Phenyl tetrazole	42.2	23.6	16.0
Benzimidazole	6.3	34.3	30.6
Colchicine	43.9	45.0	43.9
Phenyl theophylline	51.7	51.2	51.3
Acetophenone	64.1	65.1	64.1
Indole	72.1	71.5	72.1
Propiophenone	77.4	77.4	77.4
Butyrophenone	87.3	87.5	87.3
Valerophenone	96.4	96.2	96.4

**Table 2.** The protein binding data of the marketed drug molecules that were used to calibrate the retention times obtained on the chiral protein columns (Chiralpak HSA and Chiralpak AGP). The % binding data obtained by equilibrium dialysis were converted to log *k* data using log *k* = log (%binding/(101-%binding)).

Compound name	%HSA	log *k* HSA	% AGP	log *k* AGP
Warfarin	97.9	1.5	83.2	0.7
Paracetamol	14.0	-0.8	3.2	-1.5
Nizatidine	20.4	-0.6	37.1	-0.2
Trimethoprim	37.6	-0.2	46.2	-0.1
Propranolol	66.6	0.3	86.0	0.8
Carbamazepine	75.0	0.5	65.0	0.3
Nicardipine	95.0	1.2	87.0	0.8
Indomethacin	99.5	1.8	56.0	0.1
Diclofenac	99.8	1.9	60.0	0.2

**Table 3.** The calibration set of compounds used on the IAM.PC.DD2 HPLC column and their predetermined CHI IAM values.

Compound	CHI IAM
Octanophenone	49.4
Heptanophenone	45.7
Hexanophenone	41.8
Valerophenone	37.3
Butyrophenone	32.0
Propiophenone	25.9
Acetophenone	17.2
Acetanilide	11.5
Paracetamol	2.9

**Table 4.** The investigated marketed drugs, their hERG pIC_50_ values and the measured biomimetic lipophilicity CHI log *D* at pH 7.4, CHI log *P*, protein binding (log *k* HSA and log *k* AGP) and phospholipid partition (log *k* IAM). The test set of compounds are listed in the second part of the table in bold.

Drug	pIC_50_	CHI log *D*_7.4_	CHI log *P*	log *k* HSA	log *k* AGP	CHI IAM	log *k* IAM	Charge
Amitriptyline	5.10	2.65	5.21	0.91	1.05	55.27	2.96	Basic
Apomorphine	5.59	1.17	2.66	1.04	0.83	39.67	2.24	Weak Base
Astemizole	6.69	2.68	3.52	1.17	1.12	51.07	2.77	Basic
Atenolol	3.00	-0.40	0.64	-1.08	-1.83	15.75	1.14	Basic
Bepridil	6.42	4.40	6.62	1.27	1.14	57.73	3.08	Basic
Brompheniramine	5.61	1.74	4.27	0.68	0.68	51.69	2.8	Basic
Bupivacaine	5.51	3.06	3.97	-0.10	1.35	41.66	2.34	Weak Base
Carbamazepine	3.98	1.22	1.33	0.07	-0.85	23.98	1.53	Neutral
Cetirizine	4.65	1.63	2.04	0.85	0.23	40.56	2.29	Zwitterionic
Chloroquine	5.03	1.18	2.68	0.70	1.12	43.99	2.40	Basic
Chlorpromazine	5.65	-0.58	0.97	1.35	1.79	48.16	2.64	Basic
Ciprofloxacin	3.02	-0.09	0.05	-0.54	-1.19	25.39	1.59	Zwitterionic
Cisapride	6.88	2.65	3.17	1.11	0.84	41.85	2.35	Basic
Citalopram	5.27	1.59	4.32	0.40	0.44	48.80	2.66	Basic
Clarithromycin	4.32	1.9	4.55	-0.24	0.20	49.68	2.70	Basic
Clemastine	6.69	3.18	3.18	1.25	1.18	60.12	3.19	Basic
Clozapine	6.5	2.6	3.05	1.04	0.99	52.42	2.83	Weak Base
Desipramine	5.36	1.84	3.49	0.76	0.94	53.10	2.86	Basic
Diltiazem	4.81	2.76	3.12	0.36	0.41	41.95	2.35	Basic
Dolasetron	4.65	1.46	1.46	0.60	0.69	33.20	1.95	Weak Base
Domperidone	6.79	1.37	2.13	1.02	0.92	43.47	2.42	Weak Base
Doxazosin	6.03	2.09	2.24	1.04	0.72	37.80	2.16	Weak Base
Droperidol	6.89	2.26	2.68	0.98	1.69	39.31	2.23	Weak Base
Ebastine	6.27	4.96	6.54	1.62	1.34	58.65	3.12	Basic
Erythromycin	4.43	1.31	2.60	-0.22	0.32	38.79	2.20	Basic
Fexofenadine	4.61	1.28	1.91	0.45	0.02	32.24	1.90	Zwitterionic
Fluvoxamine	5.2	2.01	3.34	0.4	0.59	50.22	2.73	Basic
Glibenclamide	4.29	2.07	3.17	1.52	0.39	32.63	1.93	Acidic
Glimepiride	4.13	2.42	3.25	1.50	0.40	30.76	1.83	Basic
Granisetron	4.65	0.79	2.50	0.34	0.52	45.95	2.53	Basic
Imipramine	5.40	2.05	4.60	0.95	0.96	39.44	2.23	Basic
Isradipine	5.71	3.25	3.29	1.19	1.32	38.76	2.21	Neutral
Ketoconazole	5.49	2.66	2.84	1.18	0.75	37.86	2.16	Weak Base
Levobupivacaine	5.48	3.25	4.20	0.13	1.25	41.83	2.34	Weak Base
Levofloxacin	2.93	0.54	0.54	-0.34	-0.74	28.55	1.73	Zwitterionic
Lidocaine	3.58	2.65	3.01	-0.71	0.17	32.38	1.91	Weak Base
Lomefloxacin	3.93	-0.14	-0.14	-0.10	-1.01	37.89	2.16	Zwitterionic
Loratadine	4.90	3.86	4.25	1.38	1.34	44.30	2.46	Weak Base
Lovastatin	5.16	4.09	4.24	1.24	0.87	44.38	2.46	Neutral
Maprotiline	5.17	2.03	4.79	0.77	0.99	56.73	3.03	Basic
Mefloquine	5.45	2.18	4.22	1.38	1.11	41.65	2.29	Basic
Metoclopramide	5.27	0.5	1.91	0.15	0.23	40.38	2.28	Basic
Mibefradil	5.88	2.96	3.99	1.10	1.11	55.18	2.96	Basic
Miconazole	5.68	4.64	4.70	1.54	1.26	53.72	2.89	Weak Base
Moxifloxacin	3.83	0.69	0.81	0.54	-0.46	31.61	1.84	Zwitterionic
Nicotine	3.61	0.12	1.20	-0.03	-0.98	13.79	1.05	Weak Base
Nifedipine	3.96	2.59	2.71	0.67	0.27	25.12	1.58	Basic
Nitrendipine	5.10	3.15	3.19	1.17	0.69	39.36	2.23	Neutral
Ofloxacin	2.93	0.53	0.53	-0.40	-0.81	26.17	1.62	Zwitterionic
Olanzapine	5.06	1.96	2.76	0.76	0.72	49.74	2.71	Weak Base
Ondansetron	5.73	1.33	1.66	0.54	1.30	39.01	2.22	Weak Base
Pergolide	6.52	2.47	3.81	0.73	1.04	52.73	2.85	Basic
Perphenazine	5.88	2.92	3.50	1.39	1.13	47.64	2.61	Weak Base
Phenytoin	3.71	1.73	1.85	0.69	0.15	29.66	1.78	Weak Acid
Pimozide	6.43	3.03	3.89	1.41	1.47	52.31	2.83	Weak Base
Prazosin	5.22	1.09	1.29	0.81	0.53	26.32	1.63	Weak Base
Procainamide	3.86	-0.59	0.95	-0.71	-0.54	19.30	1.29	Basic
Propafenone	5.96	2.07	3.47	0.84	0.92	46.04	2.54	Basic
Propiverine	5.22	3.84	5.05	1.01	0.94	58.60	3.12	Basic
Propranolol	5.03	1.63	3.03	0.47	0.84	42.08	2.36	Basic
Pyrilamine	5.18	1.72	3.32	0.44	0.49	46.62	2.56	Basic
Quetiapine	5.21	2.55	2.69	0.92	0.97	39.38	2.23	Weak Base
Quinidine	5.51	1.25	2.38	0.54	0.68	49.78	2.71	Basic
Risperidone	6.00	1.46	2.18	0.55	0.63	36.31	2.09	Weak Base
Ritonavir	5.09	3.25	3.41	1.22	0.64	38.64	2.2	Neutral
Roxithromycin	4.44	1.95	3.70	-0.07	0.60	51.33	2.78	Basic
Saquinavir	5.82	3.31	3.39	1.22	1.59	42.17	2.36	Weak Base
Sotalol	3.57	-0.39	-0.35	-0.75	-1.41	21.90	1.43	Basic
Sulfamethoxazole	2.66	-0.42	1.03	0.4	-1.57	13.19	1.01	Acidic
Tamsulosin	4.89	1.43	2.01	0.36	0.60	34.32	2.00	Basic
Thioridazine	6.30	3.02	5.46	1.33	1.30	70.24	3.65	Basic
Tolterodine	6.23	2.00	4.95	0.55	0.79	52.45	2.83	Basic
Trifluoperazine	5.72	3.8	5.14	1.52	1.19	68.00	3.55	Basic
Verapamil	5.95	2.66	3.82	1.00	0.58	44.82	2.48	Basic
**Alfuzosin**	4.28	0.79	1.31	0.08	0.32	34.60	1.98	Weak Base
**Desloratadine**	5.75	1.32	4.03	0.85	1.05	54.56	2.93	Basic
**Diphenhydramine**	4.94	1.59	3.61	0.33	0.60	35.63	2.06	Basic
**Dofetilide**	5.72	1.18	1.12	-0.05	0.59	33.10	1.94	Weak Base
**Flecainide**	5.25	1.68	3.01	-0.05	0.16	41.51	2.33	Basic
**Fluoxetine**	5.73	2.15	3.80	0.99	0.99	54.86	2.94	Basic
**Indomethacin**	3.72	1.47	3.31	1.69	0.18	30.11	1.81	Acidic
**Irbesartan**	4.71	1.39	2.00	1.30	1.10	26.14	1.62	Zwitterionic
**Lamotrigine**	3.55	0.86	0.81	0.16	-0.33	23.25	1.49	Weak Base
**Metoprolol**	3.84	0.77	1.86	-0.72	-0.27	35.38	2.05	Basic
**Protriptyline**	5.30	1.72	4.70	0.69	1.03	51.59	2.79	Weak Base
**Sildenafil**	4.61	2.59	2.62	0.87	0.42	35.00	2.03	Weak Base
**Spironolactone**	4.64	2.76	2.97	0.78	0.50	36.93	2.12	Neutral
**Trazodone**	5.30	2.51	2.59	1.09	0.46	32.7	1.93	Weak Base
**Trimethoprim**	3.62	-1.60	0.42	-0.05	-0.07	12.83	1.01	Basic
**Ziprasidone**	6.44	2.98	2.96	1.42	1.09	47.92	2.62	Weak Base

**Table 5.** The calculated physicochemical properties of the compounds. MW is the molecular weight, HBD and HBA are the numbers of H-bond donor and acceptor groups, respectively, TPSA is the topological polar surface area, log *P* and log *D* are the logarithm of the calculated octanol/water partition coefficient of the neutral form and the combined ionised form of the molecules at pH 7.4, F+, F- and Fz are the calculated fractions of the positive, negative and zwitterionic charges at physiological pH (pH 7.4), A and B are the Abraham H-bond acidity and B-bond basicity parameters.

Drug	MW	HBD	HBA	TPSA	log *P*	log *D*	F+	F-	Fz	A	B
Amitriptyline	277.41	0	1	3.2	5.04	3.70	0.98	0.00	0.00	0.00	1.00
Apomorphine	267.32	2	3	43.7	2.49	2.16	0.62	0.00	0.02	0.77	1.10
Astemizole	458.57	1	5	42.3	5.70	4.54	0.98	0.00	0.00	0.13	1.64
Atenolol	266.34	4	5	84.6	0.16	-1.89	0.99	0.00	0.00	0.69	2.00
Bepridil	366.54	0	3	15.7	6.31	4.9	0.97	0.00	0.00	0.00	1.32
Brompheniramine	319.24	0	2	16.1	2.88	1.64	0.99	0.00	0.00	0.00	1.02
Bupivacaine	288.43	1	3	32.3	3.41	3.73	0.78	0.00	0.00	0.26	1.19
Carbamazepine	236.27	2	3	46.3	2.30	2.58	0.00	0.00	0.00	0.53	1.10
Cetirizine	388.89	1	5	53.0	1.70	0.34	0.00	0.22	0.78	0.57	1.76
Chloroquine	319.87	1	3	28.2	4.63	2.60	1.00	0.00	0.00	0.13	1.29
Chlorpromazine	318.86	0	2	31.8	5.35	3.34	0.99	0.00	0.00	0.00	0.94
Ciprofloxacin	331.34	2	6	72.9	-1.08	-3.03	0.05	0.03	0.91	0.73	1.85
Cisapride	465.94	3	7	86.1	4.20	2.49	0.98	0.00	0.00	0.50	2.17
Citalopram	324.39	0	3	36.3	3.76	0.99	0.99	0.00	0.00	0.00	1.08
Clarithromycin	747.95	4	14	182.9	3.16	1.16	0.95	0.00	0.00	0.80	4.49
Clemastine	343.89	0	2	12.5	5.79	3.63	0.99	0.00	0.00	0.00	0.97
Clozapine	326.82	1	4	30.9	3.32	4.68	0.58	0.00	0.00	0.18	1.44
Desipramine	266.38	1	2	15.3	4.90	1.57	1.00	0.00	0.00	0.09	0.91
Diltiazem	414.52	0	6	84.4	2.70	1.97	1.00	0.00	0.00	0.00	2.12
Dolasetron	324.37	1	5	62.4	2.70	2.66	0.10	0.00	0.00	0.31	1.52
Domperidone	425.92	2	7	67.9	4.05	3.49	0.85	0.00	0.00	0.72	1.83
Doxazosin	451.47	2	10	112.3	2.07	1.97	0.22	0.00	0.00	0.23	2.60
Droperidol	379.43	1	5	52.7	3.50	2.61	0.85	0.00	0.00	0.33	1.67
Ebastine	469.66	0	3	29.5	7.55	6.14	0.97	0.00	0.00	0.00	1.41
Erythromycin	733.92	5	14	193.9	2.54	0.65	0.95	0.00	0.00	1.05	4.63
Fexofenadine	501.65	3	5	81.0	4.35	1.83	0.00	0.04	0.97	1.20	2.12
Fluvoxamine	318.33	2	4	56.8	3.63	2.32	0.96	0.00	0.00	0.23	1.14
Glibenclamide	494.00	3	8	122.0	4.02	1.83	0.00	0.99	0.00	0.85	2.01
Glimepiride	490.62	3	9	133.1	4.25	2.05	0.99	0.00	0.00	0.75	2.15
Granisetron	312.41	1	5	50.2	0.79	-1.31	0.99	0.00	0.00	0.26	1.56
Imipramine	280.41	0	2	4.8	4.28	2.61	0.99	0.00	0.00	0.00	1.15
Isradipine	371.39	1	8	103.6	4.18	1.48	0.00	0.00	0.00	0.13	1.79
Ketoconazole	531.43	0	8	69.1	4.34	3.98	0.15	0.00	0.00	0.00	2.22
Levobupivacaine	288.43	1	3	32.3	4.35	3.73	0.78	0.00	0.00	0.26	1.19
Levofloxacin	361.37	1	7	73.3	-0.24	-2.34	0.05	0.10	0.85	0.57	2.05
Lidocaine	234.34	1	3	32.3	2.26	2.44	0.78	0.00	0.00	0.12	1.21
Lomefloxacin	351.35	2	6	72.9	-0.80	-3.43	0.05	0.03	0.91	0.73	1.81
Loratadine	382.88	0	4	42.4	5.20	4.94	0.00	0.00	0.00	0.00	1.14
Lovastatin	404.54	1	5	72.8	4.26	4.40	0.00	0.00	0.00	0.31	1.44
Maprotiline	277.41	1	1	12.0	4.85	1.48	1.00	0.00	0.00	0.13	0.68
Mefloquine	378.31	2	3	45.2	3.28	1.50	0.99	0.00	0.00	0.38	1.22
Metoclopramide	299.80	3	5	67.6	1.40	0.36	0.99	0.00	0.00	0.50	1.63
Mibefradil	495.63	1	6	67.5	4.97	2.37	1.00	0.00	0.00	0.35	1.80
Miconazole	416.13	0	3	27.1	5.34	5.45	0.15	0.00	0.00	0.00	0.79
Moxifloxacin	401.43	2	7	82.1	-0.08	-2.87	0.05	0.01	0.94	0.72	2.04
Nicotine	162.23	0	2	16.1	0.75	-0.12	0.88	0.00	0.00	0.00	0.91
Nifedipine	346.33	1	8	113.4	3.27	1.12	1.00	0.00	0.00	0.23	1.45
Nitrendipine	360.36	1	8	113.5	4.15	1.60	0.00	0.00	0.00	0.13	1.54
Ofloxacin	361.37	1	7	73.3	-0.39	-2.34	0.05	0.10	0.85	0.57	2.05
Olanzapine	312.44	1	4	59.1	3.00	4.05	0.58	0.00	0.00	0.13	1.45
Ondansetron	293.36	0	4	39.8	1.96	1.09	0.88	0.00	0.00	0.00	1.53
Pergolide	314.49	1	2	44.3	4.01	2.97	0.92	0.00	0.00	0.31	1.01
Perphenazine	403.97	1	4	55.3	3.69	3.86	0.74	0.00	0.00	0.23	1.84
Phenytoin	252.27	2	4	58.2	2.15	1.90	0.00	0.10	0.00	0.85	1.00
Pimozide	461.54	1	4	35.6	6.30	5.49	0.85	0.00	0.00	0.33	1.44
Prazosin	383.40	2	9	107.0	0.45	0.35	0.22	0.00	0.00	0.23	2.17
Procainamide	235.32	2	4	58.4	0.88	-0.83	0.99	0.00	0.00	0.50	1.45
Propafenone	341.44	2	4	58.6	3.41	1.26	0.99	0.00	0.00	0.29	1.67
Propiverine	367.48	0	4	38.8	4.01	1.53	1.00	0.00	0.00	0.00	1.31
Propranolol	259.34	2	3	41.5	3.09	0.89	0.99	0.00	0.00	0.17	1.42
Pyrilamine	285.38	0	4	28.6	3.27	1.63	0.97	0.00	0.00	0.00	1.59
Quetiapine	383.51	1	5	73.6	2.27	1.91	0.58	0.00	0.00	0.23	2.01
Quinidine	324.42	1	4	45.6	2.64	1.06	0.95	0.00	0.00	0.23	1.81
Risperidone	410.48	0	6	61.9	3.04	1.09	0.88	0.00	0.00	0.00	1.70
Ritonavir	720.94	4	11	202.3	5.64	5.64	0.00	0.00	0.00	0.88	3.14
Roxithromycin	837.04	5	17	216.9	3.79	1.00	0.95	0.00	0.00	1.05	5.12
Saquinavir	670.84	6	11	166.8	3.77	3.67	0.22	0.00	0.00	1.46	3.89
Sotalol	272.36	3	5	86.8	0.24	-1.35	0.91	0.00	0.08	0.74	1.75
Sulfamethoxazole	253.28	3	6	106.6	0.89	-0.85	0.00	0.98	0.00	0.59	1.21
Tamsulosin	408.51	2	7	119.3	2.38	1.07	0.95	0.00	0.00	0.59	2.11
Thioridazine	370.58	0	2	57.1	5.90	4.17	0.99	0.00	0.00	0.00	1.13
Tolterodine	339.51	1	2	23.5	5.98	3.49	1.00	0.00	0.00	0.50	1.08
Trifluoperazine	407.50	0	3	35.0	5.03	4.37	0.85	0.00	0.00	0.00	1.50
Verapamil	454.60	0	6	64.0	3.83	3.17	0.98	0.00	0.00	0.00	1.89
**Alfuzosin**	389.45	3	9	111.8	-0.23	-0.85	0.78	0.00	0.00	0.84	2.24
**Desloratadine**	310.83	1	2	24.9	4.13	2.42	0.98	0.00	0.00	0.13	0.99
**Diphenhydramine**	255.35	0	2	12.5	3.40	1.82	0.97	0.00	0.00	0.00	0.95
**Dofetilide**	441.57	2	8	121.6	2.41	1.06	0.88	0.00	0.08	0.72	2.16
**Flecainide**	414.34	2	5	59.6	3.78	0.32	0.99	0.00	0.00	0.41	1.32
**Fluoxetine**	309.33	1	2	21.3	4.50	2.44	1.00	0.00	0.00	0.13	0.78
**Indomethacin**	357.79	1	5	68.5	4.27	0.71	0.00	1.00	0.00	0.57	1.57
**Irbesartan**	428.53	1	7	87.1	4.72	2.22	0.00	0.00	1.00	0.56	1.78
**Lamotrigine**	256.09	4	5	90.7	2.63	2.62	0.01	0.00	0.00	0.35	0.96
**Metoprolol**	267.36	2	4	50.7	1.88	-0.43	0.99	0.00	0.00	0.17	1.76
**Protriptyline**	263.38	1	1	12.0	4.91	4.04	0.88	0.00	0.00	0.13	0.73
**Sildenafil**	474.58	1	10	117.5	2.73	2.47	0.37	0.06	0.04	0.26	2.68
**Spironolactone**	416.57	0	4	85.7	2.52	2.52	0.00	0.00	0.00	0.00	1.63
**Trazodone**	371.86	0	6	42.4	3.80	4.60	0.22	0.00	0.00	0.00	1.92
**Trimethoprim**	290.32	4	7	105.5	1.28	0.71	0.47	0.00	0.00	0.28	1.62
**Ziprasidone**	412.94	1	5	46.7	4.6	4.14	0.64	0.00	0.00	0.48	1.65

**Table 6.** Compounds selected for the test set and their indications.

Pharmaceutical	Drug class
Alfuzosin	alpha blocker
Desloratadine	tricyclic antihistamine
Diphenhydramine	antihistamine
Dofetilide	class III antiarrhythmic agent
Flecainide	class I antiarrhythmic agent
Fluoxetine	selective serotonin reuptake inhibitor (SSRI) antidepressant
Indomethacin	non-steroidal anti-inflammatory (NSAID)
Irbesartan	angiotensin receptor blocker (ARB)
Lamotrigine	antiepileptic/anticonvulsant
Metoprolol	class II antiarrhythmic agent
Protriptyline	tricyclic antidepressant
Sildenafil	phosphodiesterase (PDE) inhibitor
Spironolactone	aldosterone antagonist
Trazodone	serotonin antagonist and reuptake inhibitor (SARI) antidepressant
Trimethoprim	antibacterial
Ziprasidone	atypical antipsychotic

**Table 7.** The experimental and the predicted pIC_50_ values of the test set of compounds using the model described by equation 3. The residuals show the difference between the measured and predicted values.

Drug		Model derived with measured properties (log *k* AGP and log *k* IAM)		Model derived with calculated properties (log *P* and F-)	
	Experimental pIC_50_	Predicted pIC_50_	Residual	Predicted pIC_50_	Residual
Alfuzosin	4.28	4.74	0.46	3.85	0.42
Desloratadine	5.75	5.78	0.04	5.51	0.24
Diphenhydramine	4.94	5.02	0.08	5.23	0.29
Dofetilide	5.72	4.96	0.76	4.85	0.87
Flecainide	5.25	4.75	0.50	5.38	0.13
Fluoxetine	5.73	5.74	0.01	5.65	0.08
Indomethacin	3.72	4.55	0.83	4.08	0.36
Irbesartan	4.71	5.28	0.57	5.73	1.02
Lamotrigine	3.55	3.97	0.42	4.94	1.39
Metoprolol	3.84	4.25	0.41	4.65	0.82
Protriptyline	5.30	5.71	0.40	5.81	0.50
Sildenafil	4.61	4.85	0.24	4.89	0.28
Spironolactone	4.64	4.96	0.32	4.90	0.26
Trazodone	5.30	4.84	0.46	5.38	0.08
Trimethoprim	3.62	3.99	0.37	4.43	0.81
Ziprasidone	6.44	5.69	0.75	5.69	0.75

## References

[1] Kola I. Landis J.. Can the pharmaceutical industry reduce attrition rates?, Nat. Rev. Drug Discov. 3 (2004) 1–5. https://doi.org/10.1038/nrd1470. 10.1038/nrd1470 15286737

[2] Waring M.J. Arrowsmith J. Leach A.R. Leeson P.D. Mandrell S. Owen R.M. Pairaudeau G. Pennie W.D. Pickett S.D. Wang J. Wallace O. Weir A.. An analysis of the attrition of drug candidates from four major pharmaceutical companies. Nat. Rev. Drug Discov. 14 (2015) 475–486. https://doi.org/10.1038/nrd4609. 10.1038/nrd4609 26091267

[3] Peter Guengerich F.. Mechanisms of drug toxicity and relevance to pharmaceutical development. Drug Metab. Pharmacokinet. 26 (2011) 3–14. https://doi.org/10.2133/dmpk.DMPK-10-RV-062. 10.2133/dmpk.DMPK-10-RV-062 20978361 PMC4707670

[4] Casartelli A. Lanzoni A. Comelli R. Crivellente F. Defazio R. Dorigatti R. Fasdelli N. Faustinelli I. Pagliarusco S. Tontodonati M. Cristofori P.. A Novel and Integrated Approach for the Identification and Characterization of Drug-induced Cardiac Toxicity in the Dog. Toxicol. Pathol. 39 (2011) 361–371. https://doi.org/10.1177/0192623310390704. 10.1177/0192623310390704 21422262

[5] Ferri N. Siegl P. Corsini A. Herrmann J. Lerman A. Benghozi R.. Drug attrition during pre-clinical and clinical development: Understanding and managing drug-induced cardiotoxicity. Pharmacol. Ther. 138 (2013) 470–484. https://doi.org/10.1016/j.pharmthera.2013.03.005. 10.1016/j.pharmthera.2013.03.005 23507039

[6] O’Brien P.J.. High-content analysis in toxicology: Screening substances for human toxicity potential, elucidating subcellular mechanisms and in vivo use as translational safety biomarkers. Basic Clin. Pharmacol. Toxicol. 115 (2014) 4–17. https://doi.org/10.1111/bcpt.12227. 10.1111/bcpt.12227 24641563

[7] Ridder B.J. Leishman D.J. Bridgland-Taylor M.. A systematic strategy for estimating hERG block potency and its implications in a new cardiac safety paradigm. Toxicol. Appl. Pharmacol. 394 (2020) 114961. https://doi.org/10.1016/j.taap.2020.114961. 10.1016/j.taap.2020.114961 32209365 PMC7166077

[8] Kramer C. Beck B. Kriegl J.M. Clark T.. A composite model for hERG blockade, ChemMedChem. 3 (2008) 254–265. https://doi.org/10.1002/cmdc.200700221. 10.1002/cmdc.200700221 18061919

[9] Aptula A.O. Cronin M.T.D.. Prediction of hERG K+ blocking potency: Application of structural knowledge, SAR QSAR Environ. Res. 15 (2004) 399–411. https://doi.org/10.1080/10629360412331297353. 10.1080/10629360412331297353 15669698

[10] Raschi E. Vasina V. Poluzzi E. De Ponti F.. The hERG K+ channel: target and antitarget strategies in drug development. Pharmacol. Res. 57 (2008) 181–195. https://doi.org/10.1016/j.phrs.2008.01.009. 10.1016/j.phrs.2008.01.009 18329284

[11] Munawar S. Windley M.J. Tse E.G. Todd M.H. Hill A.P. Vandenberg J.I. Jabeen I.. Experimentally validated pharmacoinformatics approach to predict hERG inhibition potential of new chemical entities. Front. Pharmacol. 9 (2018) 1–20. https://doi.org/10.3389/fphar.2018.01035. 10.3389/fphar.2018.01035 30333745 PMC6176658

[12] Warmke J.W. Ganetzky B.. A family of potassium channel genes related to eag in Drosophila and mammals, Proc. Natl. Acad. Sci. U. S. A. 91 (1994) 3438–3442. https://doi.org/10.1073/pnas.91.8.3438. 10.1073/pnas.91.8.3438 8159766 PMC43592

[13] Du L. Li M. You Q.. The Interactions Between hERG Potassium Channel and Blockers. Curr. Top. Med. Chem. 9 (2012) 330–338. https://doi.org/10.2174/156802609788317829. 10.2174/156802609788317829 19442204

[14] Milnes J.T. Crociani O. Arcangeli A. Hancox J.C. Witchel H.J.. Blockade of HERG potassium currents by fluvoxamine: Incomplete attenuation by S6 mutations at F656 or Y652. Br. J. Pharmacol. 139 (2003) 887–898. https://doi.org/10.1038/sj.bjp.0705335. 10.1038/sj.bjp.0705335 12839862 PMC1573929

[15] Aronov A.M.. Predictive in silico modeling for hERG channel blockers. Drug Discov. Today 10 (2005) 149–155. https://doi.org/10.1016/S1359-6446(04)03278-7. 10.1016/S1359-6446(04)03278-7 15718164

[16] Redfern W.S. Carlsson L. Davis A.S. Lynch W.G. MacKenzie I. Palethorpe S. Siegl P.K.S. Strang I. Sullivan A.T. Wallis R. Camm A.J. Hammond T.G.. Relationships between preclinical cardiac electrophysiology, clinical QT interval prolongation and torsade de pointes for a broad range of drugs: Evidence for a provisional safety margin in drug development. Cardiovasc. Res. 58 (2003) 32–45. https://doi.org/10.1016/S0008-6363(02)00846-5. 10.1016/S0008-6363(02)00846-5 12667944

[17] Netzer R. Ebneth A. Bischoff U. Pongs O.. Screening lead compounds for QT interval prolongation. Drug Discov. Today 6 (2001) 78–84. https://doi.org/10.1016/S1359-6446(00)01602-0. 10.1016/S1359-6446(00)01602-0 11166255

[18] Wood C. Williams C. Waldron G.J.. Patch clamping by numbers. Drug Discov. Today 9 (2004) 434–441. https://doi.org/10.1016/S1359-6446(04)03064-8. 10.1016/S1359-6446(04)03064-8 15109948

[19] Young R.J. Green D.V.S. Luscombe C.N. Hill A.P.. Getting physical in drug discovery II : the impact of chromatographic hydrophobicity measurements and aromaticity. Drug Discov. Today 16 (2011) 822–830. https://doi.org/10.1016/j.drudis.2011.06.001. 10.1016/j.drudis.2011.06.001 21704184

[20] Fernández-Pumarega A. Martín-Sanz B. Amézqueta S. Fuguet E. Rosés M.. Estimation of the octanol-water distribution coefficient of basic compounds by a cationic microemulsion electrokinetic chromatography system. ADMET DMPK 8 (2020) 98–112. https://doi.org/10.5599/admet.760. 10.5599/admet.760 PMC8915598 35299774

[21] Bergström C.A.S. Avdeef A.. Perspectives in solubility measurement and interpretation. ADMET DMPK 7 (2019) 88–105. https://doi.org/10.5599/admet.686. 10.5599/admet.686 PMC8957229 35350542

[22] Valko K. Nunhuck S. Bevan C. Abraham M.H. Reynolds D.P.. Fast Gradient HPLC Method to Determine Compounds Binding to Human Serum Albumin. Relationships with Octanol/Water and Immobilized Artificial Membrane Lipophilicity. J. Pharm. Sci. 92 (2003) 2236-48. https://doi.org/10.1002/jps.10494. 10.1002/jps.10494 14603509

[23] Valko K. Du C.M. Bevan C.D. Reynolds D.P. Abraham M.H.. Rapid-gradient HPLC method for measuring drug interactions with immobilized artificial membrane: Comparison with other lipophilicity measures. J. Pharm. Sci. 89 (2000) 1085-96. https://doi.org/10.1002/1520-6017(200008)89:8<1085::aid-jps13>3.0.co;2-n. 10.1002/1520-6017(200008)89:8<1085::aid-jps13>3.0.co;2-n 10906732

[24] Bunally S. Young R.J.. The role and impact of high throughput biomimetic measurements in drug discovery. ADMET DMPK 6 (2018) 74–84. https://doi.org/10.5599/admet.530. 10.5599/admet.530

[25] Goetz G.H. Shalaeva M.. Leveraging chromatography based physicochemical properties for efficient drug design. ADMET DMPK 6 (2018) 71-73. https://doi.org/10.5599/admet.529. 10.5599/admet.529

[26] Hollosy F. Valko K. Hersey A. Nunhuck S. Keri G. Bevan C.. Estimation of Volume of Distribution in Humans from HPLC Measurements of Human Serum Albumin Binding and Immobilized Artificial Membrane Partitioning. J. Med. Chem. 49 (2006) 6958–6971. https://doi.org/10.1021/jm050957i. 10.1021/jm050957i 17125249

[27] Valko K. Chiarparin E. Nunhuck S. Montanari D.. In vitro measurement of drug efficiency index to aid early lead optimization. J. Pharm. Sci. 101 (2012) 4155–69. https://doi.org/10.1002/jps.23305. 10.1002/jps.23305 22930396

[28] Scheler S. Fahr A. Liu X.. Linear combination methods for prediction of drug skin permeation. ADMET DMPK 2 (2015) 199–220. https://doi.org/10.5599/admet.2.4.147. 10.5599/admet.2.4.147

[29] Stephen C. El Omri A. Ciesla L., Cellular membrane affinity chromatography (CMAC) in drug discovery from complex natural matrices. ADMET DMPK 6 (2018) 200–214. https://doi.org/10.5599/admet.535. 10.5599/admet.535

[30] Ray P.C. Huggett M. Turner P.A. Taylor M. Cleghorn L.A.T.. Spirocycle MmpL3 Inhibitors with Improved hERG and Cytotoxicity Profiles as Inhibitors of Mycobacterium tuberculosis Growth. ACS Omega 6 (2021) 2284-2311. https://doi.org/10.1021/acsomega.0c05589. 10.1021/acsomega.0c05589 33521468 PMC7841955

[31] Tsopelas F. Stergiopoulos C. Tsantili-Kakoulidou A.. Immobilized artificial membrane chromatography: From medicinal chemistry to environmental sciences. ADMET DMPK 6 (2018) 225–241. https://doi.org/10.5599/admet.553. 10.5599/admet.553

[32] Du C.M. Valko K. Bevan C. Reynolds D. Abraham M.H.. Rapid Gradient RP-HPLC Method for Lipophilicity Determination: A Solvation Equation Based Comparison with Isocratic Methods. Anal. Chem. 70 (1998) 4228-4234. https://doi.org/10.1021/ac980435t. 10.1021/ac980435t

[33] Valko K. Du C.M. Bevan C. Reynolds D.P. Abraham M.H., Rapid method for the estimation of octanol/water partition coefficient (Log Poct) from gradient RP-HPLC retention and a hydrogen bond acidity term (∑α2 H). Curr. Med. Chem. 8 (2001) 1137–1146. https://doi.org/10.2174/0929867013372643. 10.2174/0929867013372643 11472245

[34] Valko K.L. Teague S.P. Pidgeon C.. In vitro membrane binding and protein binding (IAM MB/PB technology) to estimate in vivo distribution: applications in early drug discovery. ADMET DMPK 5 (2017) 14-38. https://doi.org/10.5599/admet.5.1.373. 10.5599/admet.5.1.373

[35] Valko K.. Handbook of Analytical Separations Volume 8, Separation Methods in Drug Synthesis and Purification. 2nd ed., Elsevier 2020 p.687. ISBN: 9780444640703.

[36] Sato T. Yuki H. Ogura K. Honma T.. Construction of an integrated database for hERG blocking small molecules. PLoS One 13 (2018) 1–18. https://doi.org/10.1371/journal.pone.0199348. 10.1371/journal.pone.0199348 PMC6034787 29979714

[37] Stergiopoulos C. Makarouni D. Tsantili-Kakoulidou A. Ochsenkühn-Petropoulou M. Tsopelas F.. Immobilized artificial membrane chromatography as a tool for the prediction of ecotoxicity of pesticides. Chemosphere 224 (2019) 128–139. https://doi.org/10.1016/j.chemosphere.2019.02.075. 10.1016/j.chemosphere.2019.02.075 30818191

[38] Kaliszan R. Nasal A. Turowski M.. Quantitative structure-retention relationships in the examination of the topography of the binding site of antihistamine drugs on α1-acid glycoprotein. J. Chromatogr. A 722 (1996) 25–32. https://doi.org/10.1016/0021-9673(95)00523-4. 10.1016/0021-9673(95)00523-4 9019300

[39] Tristani-Firouzi M. Chen J. Mitcheson J.S. Sanguinetti M.C.. Molecular biology of K+ channels and their role in cardiac arrhythmias. Am. J. Med. 110 (2001) 50–59. https://doi.org/10.1016/S0002-9343(00)00623-9. 10.1016/S0002-9343(00)00623-9 11152866

